# Gut microbiota and atrial cardiomyopathy

**DOI:** 10.3389/fcvm.2025.1541278

**Published:** 2025-02-04

**Authors:** Tingting Sun, Beibei Song, Bo Li

**Affiliations:** ^1^ School of Clinical Medicine, Shandong Second Medical University, Weifang, Shandong, China; ^2^ Department of Cardiology, Zibo Central Hospital, Zibo, China

**Keywords:** atrial cardiomyopathy, gut microbiota, gut-heart axis, dysbiosis, inflammation, remodeling, short-chain fatty acids, trimethylamine n-oxide

## Abstract

Atrial cardiomyopathy is a multifaceted heart disease characterized by structural and functional abnormalities of the atria and is closely associated with atrial fibrillation and its complications. Its etiology involves a number of factors, including genetic, infectious, immunologic, and metabolic factors. Recent research has highlighted the critical role of the gut microbiota in the pathogenesis of atrial cardiomyopathy, and this is consistent with the gut–heart axis having major implications for cardiac health. The aim of this work is to bridge the knowledge gap regarding the interactions between the gut microbiota and atrial cardiomyopathy, with a particular focus on elucidating the mechanisms by which gut dysbiosis may induce atrial remodeling and dysfunction. This article provides an overview of the role of the gut microbiota in the pathogenesis of atrial cardiomyopathy, including changes in the composition of the gut microbiota and the effects of its metabolites. We also discuss how diet and exercise affect atrial cardiomyopathy by influencing the gut microbiota, as well as possible future therapeutic approaches targeting the gut–heart axis. A healthy gut microbiota can prevent disease, but ecological dysbiosis can lead to a variety of symptoms, including the induction of heart disease. We focus on the pathophysiological aspects of atrial cardiomyopathy, the impact of gut microbiota dysbiosis on atrial structure and function, and therapeutic strategies exploring modulation of the microbiota for the treatment of atrial cardiomyopathy. Finally, we discuss the role of gut microbiota in the treatment of atrial cardiomyopathy, including fecal microbiota transplantation and oral probiotics or prebiotics. Our study highlights the importance of gut microbiota homeostasis for cardiovascular health and suggests that targeted interventions on the gut microbiota may pave the way for innovative preventive and therapeutic strategies targeting atrial cardiomyopathy.

## 1 Introduction

Atrial cardiomyopathy (ACM) is a myocardial disease that primarily affects the atria. It typically presents as structural changes, dysfunction, and abnormal electrophysiological features of the atria (1–3). The etiology of ACM is complex and includes genetic factors, infection, immune dysregulation, and metabolic abnormalities. Regardless of the etiology, atrial systolic dysfunction results in atrial enlargement and fibrosis, and the clinical features of ACM are usually closely associated with atrial fibrillation and its complications, which are characterized by an increased risk of arrhythmias and stroke.

The gut microbiota (GM) is a complex ecosystem containing trillions of bacteria, viruses, fungi, and other microorganisms (4–6). These organisms play important roles in digestion, immunity, and metabolism. Metabolites from the GM can exert effects independently or through pathways involving host metabolism, thereby contributing to either the maintenance of health or the progression of disease (7), such as atherosclerotic cardiovascular disease (CVD) (8). Indeed, improving human health by modulating the biosynthesis of microbial metabolites is an emerging frontier in pharmaceutical research (9).

**Figure 1:**
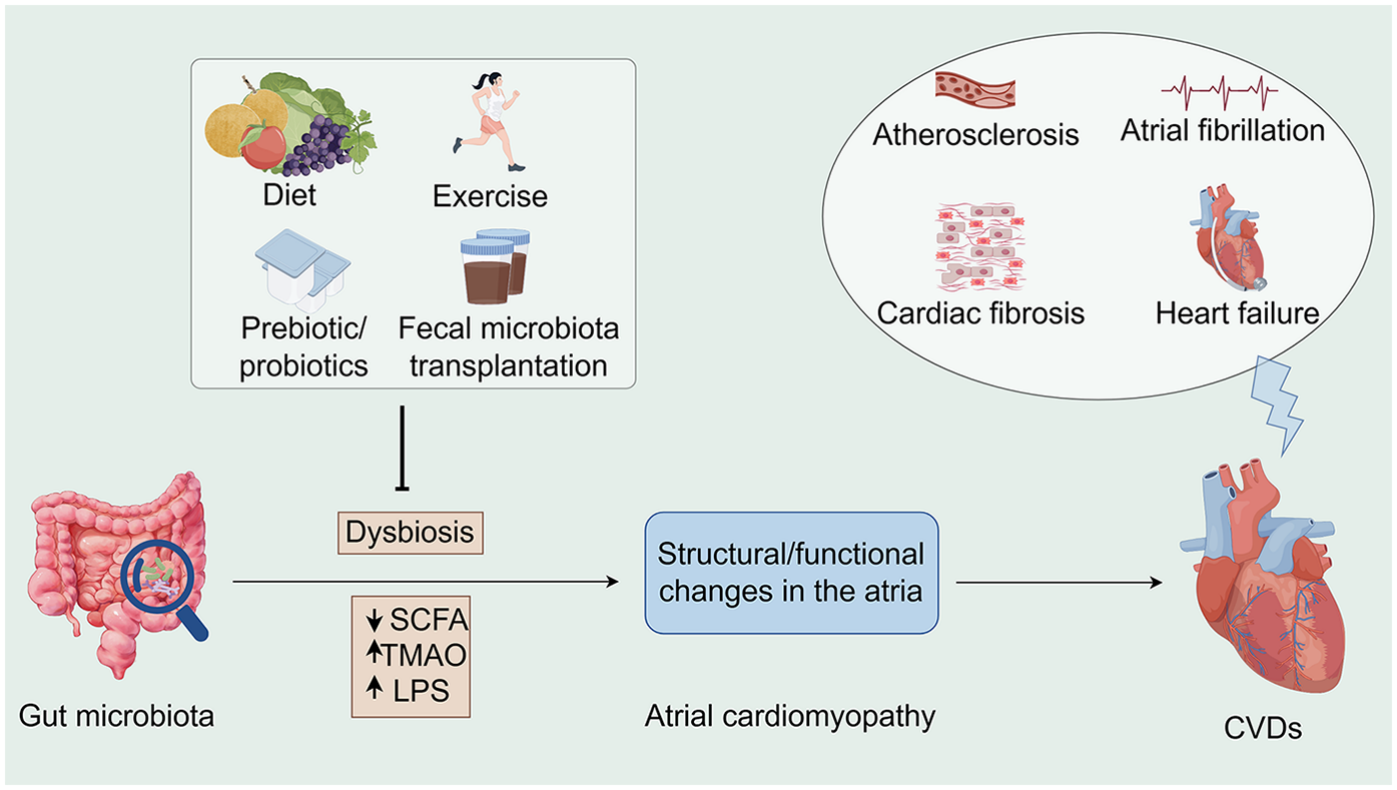
Associations between the gut microbiota, atrial cardiomyopathy, and cardiovascular diseases along potential interventions. Microbiome dysbiosis may lead to an increase in opportunistic pathogens or a decrease in SCFA-producing bacteria, which may lead to TMAO accumulation as well as a decrease in SCFA, all of which may contribute to the development of atrial cardiomyopathy and exacerbate the progression of CVDs. Conversely, interventions such as healthy diet, moderate exercise, prebiotic/probiotic use, and fecal microbiota transplantation offer opportunities to indirectly influence CVD progression. SCFA, short chain fatty acid; TMAO, trimethylamine-N-oxide; LPS, lipopolysaccharides; CVDs, cardiovascular diseases (By Figdraw).

Gut dysbiosis is a disorder of the intestinal microbiota characterized by a decrease in the number and diversity of beneficial bacteria and an increase in the number of harmful bacteria (10). This microbial imbalance has been linked to the etiology of a range of health disorders, including gastrointestinal disorders, metabolic syndrome, and cardiovascular disease (11–15). Dysbiosis can be caused by a variety of pathogenic factors, such as inadequate dietary intake, use of antibiotics, psychological stress, and chronic diseases (16). Many metabolites, such as short-chain fatty acids (SCFAs) (17, 18), bile acids (19, 20), and trimethylamine N-oxides (TMAO) (21–23), are affected by gut microbial–host interactions, which in turn affect intestinal health and function, as well as various metabolic pathways in the host (24–26) (Figure 1).

ACM is a disease that affects the structure and function of the heart. In recent years, research has focused on the relationship between gut health and heart disease. Dysbiosis of the gut microbiota may influence the development of ACM through several mechanisms: inflammatory responses, changes in metabolites, and effects of the immune system. Certain circulating metabolites may increase the risk of cardiovascular events and subsequent pathological responses due to changes in the microbiota (27, 28). Several studies have confirmed the existence of a gut–heart axis (29, 30). Thus, the relationship between the GM and underlying cardiac disease has been well studied. However, the relationship between the GM and the cardiac pathological condition ACM has not been characterized to date. To address this issue, we review the existing literature and discuss the role of the GM in the occurrence and development of ACM. Based on our findings, modification of the microbiota appears to be a potential therapeutic approach to prevent the progression of ACM.

## 2 Pathophysiology of atrial cardiomyopathy

### 2.1 Changes in atrial structure and function

#### 2.1.1 Changes in atrial structure

Atrial hypertrophy, fibrosis, and remodeling are the major morphologic changes observed in the atrial structure (31–33). Atrial fibrosis is invariably associated with atrial hypertrophy, which is induced by prolonged pressure or volume overload and ultimately leads to proliferation and hypertrophic dilatation of atrial myocytes (34, 35). Atrial fibrosis is a major feature of ACM and manifests as an abnormal accumulation of collagen within the atrial myocardium (16, 33). This pathological deposition leads to changes in the electrophysiological properties of the atria and increases susceptibility to arrhythmias (36). In addition, as the disease progresses, the atria undergo significant geometric and structural changes, including atrial wall thickening and ventricular dilatation, which may affect overall cardiac function (37).

In addition to atrial enlargement, atrial wall fibrosis, and the presence of atrial anatomic abnormalities (1, 2), other structural changes occur in ACM, including atrial trabecular hyperplasia, atrial microarteriovenous atheromatosis, and intra-atrial thrombosis (38–40). Together, these changes contribute to the complex pathophysiology of ACM, highlighting the need for a comprehensive understanding of the multiple effects of the disease on cardiac structure and function.

#### 2.1.2 Changes in atrial function

The atria play both active and passive roles in the cardiac cycle, which can be divided into three distinct phases: the atrial storage phase, the atrial conduction phase, and the atrial contraction phase. During the atrial storage phase, the atria accommodate venous return by adjusting the interval between closure and opening of the atrioventricular (AV) valve during ventricular systole. The ability of the atria to store blood depends on atrial compliance and diastolic function (41). Simultaneously, as intra-atrial pressure rises and the ventricles actively relax, the atria enter the atrial conduit phase with the onset of AV valve opening. During the initial phase of ventricular diastole, blood from the atria, systemic veins, and pulmonary veins passively fills the ventricles, while the atria play the role of conduit. The pressure gradient between the atria and the ventricles gradually decreases, causing ventricular filling to slow or stop in mid-diastole, a phase known as diastole. Finally, at the end of ventricular diastole, the atria actively push the remaining blood into the ventricles by contracting. In healthy individuals, the atria contribute approximately 40%, 35%, and 25% to ventricular filling during the blood storage, inflow, and contraction phases, respectively (42). In atrial fibrillation (AF), ventricular filling is more dependent on the conduit phase because of the loss of systolic function and the significant reduction in blood storage due to atrial stiffness (43).

The main functional changes in ACM are manifested by altered electrophysiological properties and impairment of atrial systolic and diastolic function. ACM induces electrophysiological abnormalities, such as decreased electrical conduction velocity and increased autoregulation, which can lead to electrical instability of the atria and an increased tendency to develop atrial fibrillation and other arrhythmogenic events (1, 32, 41, 44).

In addition, atrial contractility is often impaired in ACM due to atrial myocyte dysfunction and dynamic changes in intra-atrial pressure. This dysfunction reduces the ability of the atria to push blood during ventricular contraction. At the same time, diastolic atrial function is also impaired, as evidenced by the inability of the diastolic atria to adequately fill with blood (1, 40, 43). This deficiency leads to an increase in intra-atrial pressure, which exacerbates structural remodeling of the atria.

These functional abnormalities highlight the intricate interplay between electrophysiological and mechanistic alterations in atrial electrophysiology and mechanics in the pathogenesis of ACM, and a nuanced approach is required to understand and manage this complex heart disease.

### 2.2 Cellular and molecular mechanisms of atrial cardiomyopathy

The electrophysiologic characteristics of ACM are closely related to a range of cellular and molecular mechanisms. These mechanisms include the electrophysiological properties of atrial myocytes, intercellular electrical coupling, and the process of atrial remodeling. Atrial myocytes in patients with ACM can exhibit different electrophysiological characteristics (1, 38, 45), such as changes in action potential duration and alterations in ion channel function. These changes may affect atrial excitability and conduction, thereby inducing arrhythmias. The presence of atrial fibrosis in ACM leads to reduced intercellular electrical coupling. Reduced coupling leads to heterogeneous propagation of electrical signals within the atria, which in turn leads to instability of electrical activity within the atria and an increased risk of reentrant cardioversion excitation (35, 38, 44–46). ACM is closely associated with atrial remodeling (47), which is a multifaceted process involving structural changes, apoptosis, and the initiation of fibrosis. During the remodeling process, the electrophysiological characteristics of atrial myocytes undergo progressive changes, ultimately leading to deterioration of atrial electrophysiological function and subsequent arrhythmias (1, 38, 40, 44, 46).

In summary, the electrophysiological features of ACM are central to a complete understanding of the disease. These features include a shortened effective atrial refractory period, delayed atrial conduction, and increased reentrant excitation, all of which are closely associated with alterations in atrial structure and function (39). By elucidating these electrophysiological features and their underlying cellular and molecular mechanisms, clinicians can more accurately assess and reduce the risk of arrhythmias in patients with ACM.

### 2.3 Signaling mechanisms in atrial cardiomyopathy

**Figure 2:**
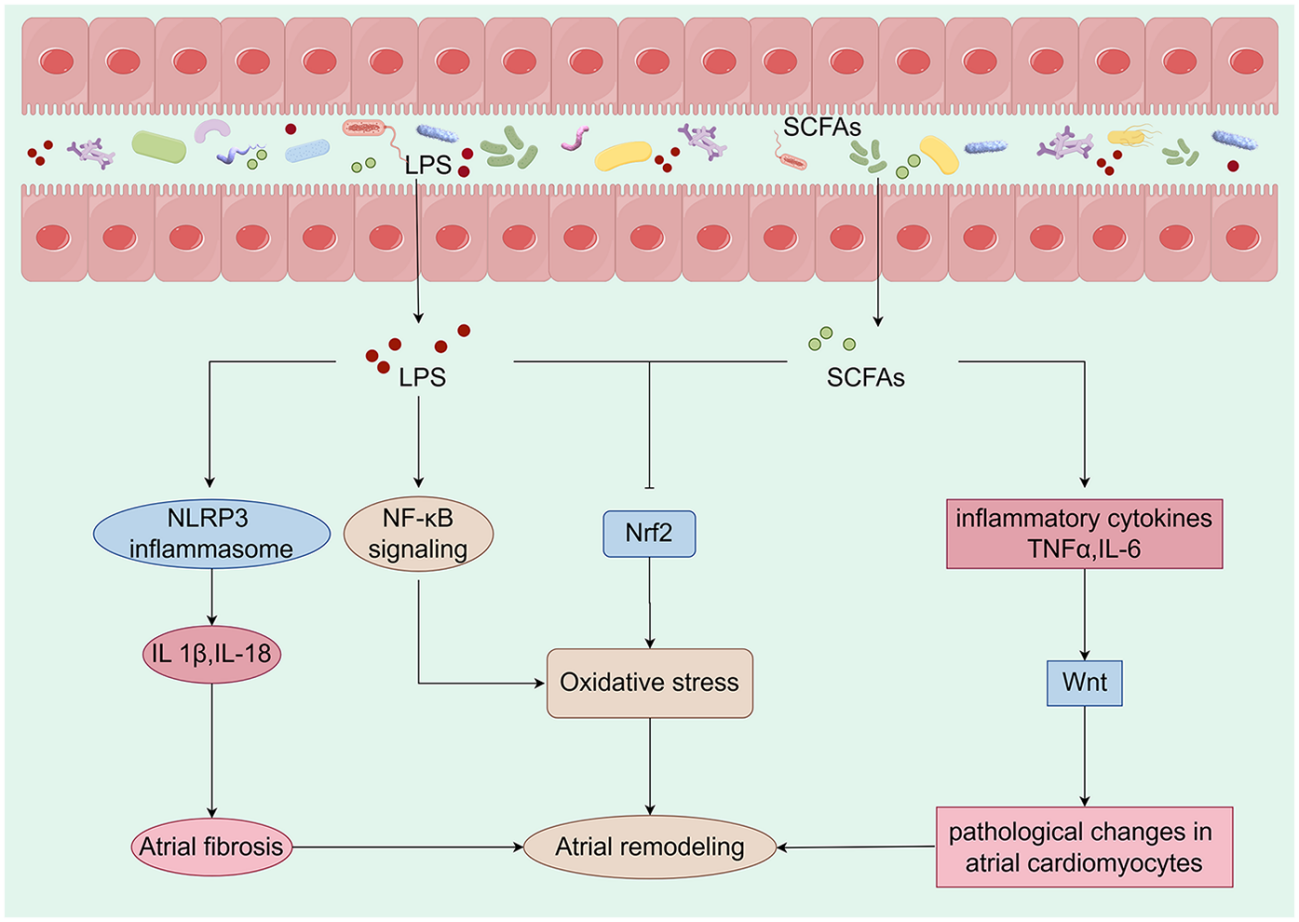
Metabolites derived from the gut microbiota are closely linked to mechanisms that promote atrial remodeling. Alterations in the composition of the gut microbiota can initiate changes in metabolic pathways, which may lead to the translocation of bacteria, their fragments, and products into the circulation. This process can intensify the pro-inflammatory environment and induce metabolic dysregulation, creating a conducive setting for the development of cardiovascular disease. Specifically, LPS activates the NLRP3 inflammasome, resulting in the production of IL-1β and IL-18, which contribute to atrial fibrosis. Furthermore, LPS may enhance oxidative stress by activating the NF-κB pathway or inhibiting the expression of Nrf2. A reduction in the production of SCFAs may lead to an overproduction of TNF-α and IL-6, inflammatory factors that activate the Wnt pathway and induce pathological changes in atrial cardiomyocytes. Concurrently, the decrease in SCFAs may also inhibit Nrf2 production, further exacerbating oxidative stress and promoting atrial remodeling. Collectively, these metabolites derived from gut microbiota have a significant impact on atrial electrophysiology and structural remodeling. LPS, lipopolysaccharide; SCFAs, short-chain fatty acids; IL-1β, interleukin-1 beta; IL-6, interleukin-6; IL-18, interleukin-18; TNF-α, tumor necrosis factor-alpha (By Figdraw).

The development of ACM is closely related to the electrophysiological activity of the atria, particularly the atrial remodeling process. The atria play an important role in cardiac function, including the regulation of left ventricular filling pressures and cardiovascular function. Structural and functional changes in the atria affect these physiological processes and contribute to the development of ACM (Figure 2).

#### 2.3.1 Wnt signaling pathway

The Wnt pathway plays an important role in ACM. Studies have shown that activation of the Wnt pathway is associated with the development of ACM (48, 49). This pathway influences the structural remodeling and electrophysiological properties of the atria by regulating cell proliferation and differentiation (50, 51). The GM influences the host's physiological state through its metabolic products, particularly SCFAs (52–54). These SCFAs modulate immune responses and inflammation levels, thereby indirectly affecting the Wnt signaling pathway (55, 56). Research indicates that certain SCFAs may promote cardiac cell proliferation and repair by regulating the Wnt signaling pathway (57–59). Dysbiosis of the GM is often associated with chronic low-grade inflammation, which can activate the Wnt signaling pathway, leading to pathological changes in atrial cardiomyocytes (60). Specifically, inflammatory cytokines such as tumor necrosis factor-alpha and interleukin-6 (IL-6) can enhance the transmission of Wnt signals through various pathways, thereby promoting the progression of ACM (61).

#### 2.3.2 NLRP3 inflammasome pathway

The NLRP3 inflammasome is a critical component of the immune response, capable of detecting intracellular pathogens and damage signals (62, 63). Dysbiosis of the GM leads to elevated levels of lipopolysaccharides (LPS), which activate the NLRP3 inflammasome via Toll-like receptors (64, 65). The activation of the NLRP3 inflammasome results in the release of pro-inflammatory cytokines, such as interleukin-1 beta (IL-1β) and interleukin-18 (IL-18), which contribute to atrial remodeling and electrophysiological abnormalities (66–68). Furthermore, the release of these cytokines promotes atrial fibrosis, thereby increasing the risk of AF (69, 70). Similarly, an imbalance in the GM can impair cardiac function and elevate the risk of cardiovascular events (71, 72).

#### 2.3.3 Nrf2 pathway

ACM is a pathological condition characterized by alterations in atrial structure and function, frequently associated with AF and heart failure (HF) (73, 74). Nrf2 is a pivotal transcription factor that regulates antioxidant responses and maintains cellular homeostasis. In the context of cardiovascular health, Nrf2 plays a critical role by mitigating oxidative stress and inflammatory responses, thereby protecting cardiac function (75–77). Oxidative stress has been shown to significantly contribute to the development of atrial cardiomyopathy (78–80). As oxidative stress levels increase, calcium homeostasis in cardiac cells is disrupted, leading to changes in the electrophysiological properties of the atrium and the initiation of arrhythmias (81–83). Dysbiosis of GM may promote the progression of ACM through several pathways: 1. Dysbiosis can lead to increased intestinal permeability, allowing endotoxins to enter the circulation and activate a systemic inflammatory response. This inflammatory state may further exacerbate oxidative stress by activating NF-κB and other pathways while down-regulating Nrf2 (84–86). 2. Metabolites such as SCFAs, produced by gut microbes, have protective effects on heart health. However, dysbiosis leads to a reduction in these beneficial metabolites, impairing Nrf2 activation and promoting the development of atrial cardiomyopathy (87, 88). 3. Autonomic nervous system regulation: The GM and its metabolites may influence the cardiac autonomic nervous system through direct or indirect mechanisms, subsequently affecting the electrophysiological characteristics of the heart. This may occur by modulating Nrf2 expression and activity (86, 89, 90).

#### 2.3.4 Key molecules and their roles

Several key molecules play important roles in the cellular and molecular mechanisms of ACM that affect atrial structure and function. These molecules include cyclooxygenase-2 (COX-2), endothelin-1 (ET-1), and other factors (91) that play key roles in cardiac remodeling, atrial dysfunction, vasoconstriction, and inflammatory responses.

##### 2.3.4.1 Cyclooxygenase-2 (COX-2)

COX-2 is a key enzyme in prostaglandin biosynthesis and is involved in a variety of physiological and pathological processes. Its functions are manifold and include the following three factors. (1) The inflammatory response: COX-2 is upregulated in response to inflammation and its expression level is significantly increased in patients with ACM. It mediates the local inflammatory response through the synthesis of prostaglandins, which affect atrial function and morphology (92, 93). (2) Cardiac remodeling: COX-2 expression is increased in conditions such as myocardial infarction and HF, leading to increased cardiac remodeling and functional decline. Pharmacological inhibition of COX-2 has been shown to improve cardiac function, making it a promising target for therapeutic intervention (94, 95). (3) Apoptosis: COX-2 activity is associated with cardiomyocyte apoptosis, which in turn promotes atrial fibrosis and dysfunction.

In summary, COX-2 has a major impact on the pathogenesis of myocardial ischemia through its involvement in inflammation, cardiac remodeling, and apoptosis, highlighting its potential as a therapeutic target to mitigate disease progression (96).

##### 2.3.4.2 Endothelin-1 (ET-1)

ET-1 is a potent vasoconstrictor with important effects on the heart and blood vessels. Its major functions are as follows. (1) Vasoconstriction: By binding to endothelin receptors, ET-1 induces vascular smooth muscle contraction and elevated blood pressure (97–99), which plays an important role in the pathophysiology of ACM. (2) Cardiac remodeling: ET-1 promotes cardiomyocyte proliferation and fibrosis, which leads to structural changes in the atria, triggering atrial dysfunction and AF (100). (3) Promotion of the inflammatory response: ET-1 is also involved in regulation of the inflammatory response and can stimulate the release of cytokines that exacerbate atrial damage and fibrosis (101).

##### 2.3.4.3 Other key molecules

In addition to COX-2 and ET-1, a number of other important molecules are involved in ACM as follows. (1) Neurotrophic factors, such as brain-derived neurotrophic factor, which play an important role in adaptive remodeling of the heart and affect cardiomyocyte survival and function (102, 103). (2) Cytokines, such as tumor necrosis factor-alpha (TNFα) and IL-6, which can contribute to inflammation and apoptosis in ACM, further exacerbating cardiac dysfunction (104). (3) Atrial natriuretic peptide, a hormone secreted by the atria with diuretic and antihypertensive effects that reduces atrial workload and improves cardiac function (105–107).

## 3 Brief overview of the gut microbiota

Sequencing technology has advanced dramatically over the past decade, allowing researchers around the world to assess how genetic modifications affect human health. Humans develop symbiotic relationships with microorganisms from an early age (108). Factors such as environment (109, 110), proximity to other humans and animals (111), diet (112, 113), genetics (114–116), and temporal changes (117–119) influence the microbial communities in our skin, mouth, and gut (120, 121). In terms of its impact, the GM has been likened to a previously unknown organ; it is extensively metabolized and carries 150 times more genes than the human genome, providing the host with a range of metabolic capabilities that would otherwise be unavailable (122). Unlike the human genome, the GM is relatively plastic. It can be rapidly altered by factors such as diet, drugs, probiotics, and metabolites produced by microbes. Therefore, intentional modification of the GM may have health consequences. Transgenesis is increasingly recognized as an important target for drugs, and certain microbes have been shown to inactivate or activate certain exogenous substances, thereby altering the effects of various therapeutic drugs (123). We are just beginning to understand the systemic effects of whole transgenes on the entire metabolite pool.

Studies have shown that a healthy GM correlates with high microbial diversity and abundance (124–128), which is largely influenced by the host's diet, lifestyle, and genetic predisposition. GM diversity is typically assessed using two parameters, namely species richness and species evenness. A robust gut may contain approximately 1,000 different bacterial species (129, 130), with inter-individual variation attributable to dietary, environmental, and genetic factors. Under healthy conditions, GMs are characterized by a relatively even distribution of bacterial species; however, under pathological conditions such as inflammatory bowel disease or ACM, certain species may overgrow, leading to dysbiosis (131). Transgenic diversity affects not only digestive and metabolic processes, but also the immune system, neuroendocrine regulation, and the cardiovascular health of the host (132).

The composition and function of the GM is critical to the maintenance of overall health. It is closely linked to nutrient metabolism, immunomodulation, and pharmacokinetics, and is essential for maintaining the integrity of the GM. Dysregulation of the GM can lead to a number of health problems, particularly affecting cardiovascular health (131).

## 4 Intestinal dysbiosis and atrial cardiomyopathy

It is hypothesized that gut dysbiosis has multiple effects on the etiology and progression of ACM through the following mechanisms. (1) The inflammatory response: Gut dysbiosis is often associated with increased systemic inflammation. Bacterial metabolites (e.g., lipopolysaccharides) can cross the intestinal barrier as a result of increased permeability, triggering a systemic inflammatory cascade that leads to cardiac tissue damage and the progression of ACM (133, 134). (2) Effects of metabolites: Gut microbial metabolites such as SCFAs have anti-inflammatory and cardioprotective properties. Interfering with genetic modifications may reduce SCFA production, which may decrease cardioprotective effects and increase susceptibility to acute myocardial infarction (135, 136). (3) Dysfunction of the intestinal barrier: Dysbiosis of the transgenic microbiota induces intestinal epithelial dysfunction, which increases intestinal permeability and facilitates the translocation of endogenous toxins and bacteria into the systemic circulation. This translocation can trigger an inflammatory response that may lead to the development of ACM (133, 137, 138). (4) Microbiota diversity: Empirical evidence suggests that patients with HF have reduced microbiota diversity, which may be associated with the development of ACM. Genomes with reduced diversity may be less effective in maintaining gut and immune health, thereby increasing the risk of cardiovascular disease (139, 140).

The interactions between gut dysbiosis and ACM are complex and involve inflammatory responses, metabolite synthesis, gut barrier integrity, and microbial diversity. Enhancing microbial diversity and metabolic function by modulating the gut microbiota may be a strategy to reduce the risk of ACM.

### 4.1 Impact of gut dysbiosis on atrial cardiomyopathy and its underlying mechanisms

In recent years, an increasing number of studies have demonstrated a significant correlation between the composition of the GM and its metabolites and the incidence of a variety of cardiovascular diseases, with a particular focus on ACM (8, 9, 16, 141), a cardiac disorder characterized by structural and functional changes in the atria that can lead to severe arrhythmias, including AF. The relationship between the GM and AF remains to be elucidated (142, 143). The hypothesis is that GM or its metabolic by-products have effects on distal cellular targets, as indicated by the presence of key metabolites. These include SCFAs (the major end products of microbial fermentation of dietary fiber), TMAO, and lipopolysaccharides (26, 144, 145).

The left atrium (LA) serves three primary functions: fluid storage, conduit, and contraction. The interplay among these functions is essential for optimal ventricular filling and cardiac output, with the LA often being the first to respond to left ventricular (LV) diastolic dysfunction. Over time, however, the LA loses its contractile function, leading to mechanical failure and structural changes (146). Patients with LV diastolic dysfunction in the preclinical phase of HF have an abnormal LA strain and left atrial volume index, although the prevalence of abnormal strain is generally high (147). The dependence on the contribution of the LA to LV filling increases as LV diastolic dysfunction progresses (148). Some patients eventually progress to the clinical stage of HF. The presence of abnormal left strain during the reservoir phase is independently associated with incident HF despite a normal left atrial volume index (149). The risk of HF events associated with LA structural and functional abnormalities is not related to the left ventricular ejection fraction or natriuretic peptide levels (150). Alterations in atrial mechanics, particularly the LA, play an important role in various aspects of HF with a preserved ejection fraction. Emerging evidence suggests that gut ecological dysregulation has an impact on clinical HF and its subtypes (e.g., HF with a preserved ejection fraction) (151). Although the underlying mechanisms of the gut–heart axis during HF remain largely unknown, increased filling pressures and impaired diastolic blood pressure, which may lead to a progressive decrease in cardiac output, have been proposed as main drivers of gut ecological dysregulation (152). These changes in gut composition are characterized by a decrease in microbial α-diversity and a decrease in the number of beneficial bacteria, such as those with the potential to produce SCFAs (151). At the same time, the number of pathogenic bacteria in the gut ecosystem increases. In addition, gut dysbiosis has been shown to affect human health by modulating the host's circulating metabolite profile. This has been attributed to the ability of the GM to produce a wide range of functional metabolites that can enter the circulation alone or in concert with host metabolic processes (153).

Gut dysbiosis leads to reduced SCFA production, which triggers metabolic dysregulation and systemic inflammation, both of which are potential risk factors for the development of ACM.

With the development of gut dysbiosis, intestinal barrier function is compromised, allowing endogenous bacterial components (e.g., lipopolysaccharides) to enter the circulation and trigger a systemic inflammatory response (15). Studies have shown that chronic inflammation is closely associated with the development of atrial cardiomyopathy. Elevated levels of inflammatory factors such as TNF-α and IL-6 may damage atrial myocytes and remodel the atrial structure, leading to the development of ACM (104).

In addition to affecting the host immune response, GM also affects cardiac function through the production of various metabolites. For example, some metabolites produced by the GM, such as TMAO (20–22), have been shown to be associated with an increased risk of cardiovascular disease, and accumulation of TMAO may indirectly affect the electrophysiological properties of the atria by promoting atherosclerosis and myocardial damage, leading to ACM (15).

Changes in metabolites due to gut dysbiosis affect the electrophysiological properties of the heart (154). Studies have shown that metabolites produced by the GM affect the electrical activity of the heart, leading to changes in cardiac autoregulation and excitability. These changes can lead to atrial myocardial remodeling and electrical conduction abnormalities, increasing the risk of AF and other arrhythmias (8, 15).

To prevent ACM, it is important to maintain a good glycemic balance. This can be achieved through a healthy diet (high in fiber, low in sugar, high in prebiotics and probiotics) (126, 127, 151), moderate exercise, and good lifestyle habits. By promoting the growth of beneficial microorganisms, intestinal barrier function can be improved and systemic inflammatory responses can be reduced, which in turn promotes cardiovascular health.

### 4.2 Role of the gut–cardiovascular axis in atrial cardiomyopathy

The gut–heart axis is a biological mechanism that describes how the GM affects heart health through multiple pathways (155). Studies have shown that the GM may interact with the heart in the following ways. (1) The inflammatory response: Gut dysbiosis leads to increased intestinal permeability, which allows endogenous toxins and bacterial metabolites to enter the bloodstream and trigger systemic inflammation, which is thought to be an important mechanism in cardiovascular disease (133). (2) Metabolites: The GM produces SCFAs and other metabolites that may affect cardiac metabolism and function (156, 157). (3) Neurological signaling: The GM interacts with the central nervous system via the vagus nerve and affects autonomic regulation of the heart (158).

Gut dysbiosis is strongly associated with the development of ACM (16). Reduced GM diversity can lead to inflammation and fibrosis of cardiac tissue, which in turn can lead to atrial dysfunction (159, 160). Gut dysbiosis is usually associated with chronic low-grade inflammation, and this inflammatory state may promote electrophysiological remodeling of the atria, increasing the risk of AF. Changes in GM composition can strongly influence the synthesis of metabolites, and TMAO is one such metabolite that has been extensively studied for its role in increasing the risk of cardiovascular disease. The mechanisms of action of TMAO are diverse and include modulation of tissue sterol metabolism (26, 161, 162), which may alter cholesterol distribution and metabolism; enhancement of endothelial cell activation, which promotes vascular inflammation (26, 163–165); and stimulation of the pro-fibrotic pathway (166), which may contribute to pathological remodeling of cardiovascular tissue. These metabolites may affect the heart through a variety of mechanisms, including improvement of endothelial function and suppression of inflammatory responses. The gut–heart axis also includes transgenes that may affect heart health through neural mechanisms. Studies have shown that the GM can affect autonomic homeostasis in the heart by modulating vagal activity, which may play a role in ACM (167).

### 4.3 Correlation between gut dysbiosis and atrial cardiomyopathy

A growing number of studies have examined the association between the GM and metabolic and CVD, including coronary heart disease and HF (168, 169). Macrogenomic analyses of various CVD patient populations have demonstrated significant differences in GM composition in the presence or absence of CVD and HF (170, 171). In addition, metabolomics-based clinical studies (172, 173) and mechanistic studies in animal models have further confirmed the potential causal role of the genome in the development of CVD and hyperlipidemia (174–176). GM produces a diverse array of metabolites, including SCFAs, amines, and phenolic compounds. These metabolites significantly contribute to the pathophysiology of ACM through multiple pathways: they modulate immune responses, alter cardiac metabolism, and interact with receptors in cardiac tissue (177).

In clinical observations, many studies have found significant differences in the GM composition of AF patients compared with healthy individuals. It has been established that the metabolic interactions between the gut and the host play a pivotal role in the development of AF (178). For example, it has been shown that certain GM compositions are significantly decreased in AF patients, while others are increased, which may be related to the pathogenesis of AF (179, 180). Dysregulation of the GM has been demonstrated to result in alterations to cardiac structure and function, thereby elevating the risk of AF (181). In addition, changes in GM are strongly correlated with a patient's cardiac functional status and metabolic profile, suggesting that gut health may be potentially valuable in the prevention and treatment of ACM. Research indicates that GM and its metabolites may significantly influence the development of HF. Patients diagnosed with HF often exhibit a pronounced imbalance in their GM, which may be closely associated with the pathophysiological mechanisms underlying ACM (182). Changes in GM not only affect the direct symptoms of ACM but may also indirectly contribute to the onset of heart failure by modulating cardiovascular risk factors, including metabolic syndrome, obesity, and diabetes (177, 183, 184).

Laboratory studies also support a link between the GM and ACM. For example, a study in an animal model found that modulating genetic changes improved ventricular function and reduced the risk of developing atrial myopathy. Specifically, antibiotic intervention significantly improved atrial structure and electrophysiological properties in an animal model, suggesting that changes in the GM may directly affect cardiac physiology (185).

Currently, numerous clinical studies and fundamental experiments are in progress to elucidate the precise mechanisms by which GM and its metabolites contribute to ACM (21). Researchers have employed a variety of advanced technologies, including high-throughput sequencing and metabolomics analysis, to gain a comprehensive understanding of the interactions between GM and cardiovascular health.

Future research should focus on enhancing outcomes for patients with ACM by modulating GM through interventions such as probiotics or dietary modifications. This strategy represents a novel therapeutic approach with significant potential for clinical applications (186, 187).

## 5 Interventions for atrial cardiomyopathy

### 5.1 Dietary changes

Fiber is an important source of energy for the body. Increased intake of whole grains, legumes, fruits, and vegetables may improve heart health by promoting the growth of beneficial bacteria and increasing the production of SCFAs. Epidemiologic studies have shown that adequate fiber intake helps prevent dyslipidemia and atherosclerotic vascular disease (188). Omega-3 fatty acids have anti-inflammatory properties and can be obtained from foods such as fish, nuts, and seeds (189). These fatty acids are not only good for the heart, but also help improve the composition of the blood. Adequate hydration helps maintain a healthy digestive system and promotes gut microbial balance (190).

### 5.2 Lifestyle changes

Lifestyle modifications can significantly slow the progression of ACM by influencing the GM and its metabolites (191–194). Engaging in moderate exercise and ensuring adequate sleep can promote the growth of beneficial microbes, thereby enhancing heart health. Regular physical activity has been shown to improve GM diversity and foster the proliferation of advantageous bacteria. Research indicates that exercise enhances gut barrier function and reduces systemic inflammation, both of which contribute to slowing the progression of ACM (195–198). Additionally, exercise increases the heart's pumping capacity and electrophysiological stability, thereby lowering the risk of arrhythmias associated with ACM (199–202). Conversely, sleep deprivation is linked to an elevated risk of various cardiovascular diseases, while quality sleep facilitates physical repair and supports immune function (203, 204). Studies have demonstrated that improved sleep quality can help restore GM balance and mitigate inflammatory responses, which is beneficial for managing ACM (205, 206).

### 5.3 Use of probiotics and prebiotics

Probiotics are live microorganisms that are beneficial to the health of the host (207). Common probiotics include *Lactobacillus* and *Bifidobacterium*, and supplementation with these probiotics may help to restore the GM balance. Prebiotics are food ingredients that promote the growth of beneficial microorganisms, such as inulin and oligofructose. Supplementation with prebiotics may increase the number and activity of beneficial bacteria in the gut (208). Probiotics are typically administered through oral supplements or fermented foods, both of which are user-friendly and well-accepted by patients (182, 209, 210). They play a crucial role in regulating intestinal microbiota, enhancing intestinal barrier function, and reducing intestinal inflammation, thereby contributing to overall health improvement (211, 212). Research has indicated that specific probiotics may positively influence cardiovascular health by lowering blood pressure and improving lipid profiles, which could indirectly benefit patients with ACM (213). In comparison to pharmacological treatments, probiotics generally exhibit fewer side effects, which are often benign and self-limiting (214). However, the health effects of various probiotic strains can differ significantly, and some may have limited effectiveness in ameliorating ACM (215, 216). Consequently, selecting appropriate probiotics is essential. The market offers a wide range of probiotic products; however, the absence of uniform quality control and standardization can result in variable efficacy (217). Furthermore, the benefits of probiotics often necessitate long-term usage to sustain their effects, which may pose convenience challenges for patients requiring consistent dietary supplementation (218). Although preliminary studies suggest potential benefits of probiotics on cardiovascular health, there remains a lack of robust clinical evidence supporting their application in ACM, necessitating further research to confirm their efficacy (219).

### 5.4 Microbiota transplantation

Fecal microbiota transplantation is an emerging therapy that restores the diversity and function of gut microbes by transplanting the GM from healthy donors into patients (220–223). FMT has been shown to effectively restore the intestinal microbiome in patients, thereby correcting gut dysbiosis (186, 224–226). This restoration may alleviate systemic inflammatory responses, potentially benefiting cardiovascular health. Several studies indicate that FMT can reduce myocardial damage and improve cardiac function by reestablishing a healthy microbiota (21, 227). This is particularly significant for patients with ACM, as enhanced atrial function could decrease the incidence of AF (178, 228). FMT may exert its effects by modulating the immune system, which plays a crucial role in cardiovascular diseases where immune-inflammatory responses are among the key pathophysiological mechanisms. The efficacy of FMT can be optimized through personalized adjustments based on an individual's unique microbiome profile, thereby enhancing therapeutic outcomes (224, 229). However, FMT involves the transplantation of fecal matter from healthy donors to patients, which poses risks of infectious disease transmission (186). Although stringent screening protocols are implemented, caution remains essential. The effectiveness of FMT varies among individuals, and some patients may not experience significant improvements. Additionally, the duration of therapeutic effects can be limited, with some patients experiencing relapses shortly after treatment (186). The acceptability of FMT may be challenged due to its association with fecal matter, both among patients and healthcare providers (186). Ethical concerns may also impede its widespread adoption and application (230). Currently, there is limited research on the long-term effects and safety of FMT in patients with atrial cardiomyopathy, underscoring the need for large-scale clinical trials to validate its sustained efficacy (231).

## 6 Conclusions

Gut dysbiosis, recognized as a significant correlate of several diseases, has emerged as a prominent factor in ACM. It is involved in the systemic inflammatory profile of the host and modulates the oxidative state via the gut–heart axis. The influence of the GM on systemic health through the regulation of immune responses, inflammatory mediators, metabolic pathways, and nervous system function is now widely recognized as substantial and should not be overlooked. Consequently, maintaining a diverse and balanced GM is essential for optimal health, with potential preventive and therapeutic implications for certain diseases. However, the precise mechanisms by which the GM exerts its influence on health require further elucidation, particularly in the context of probiotic interventions. Current research into the interplay between heart disease and the GM is predominantly limited to animal models, with a paucity of large-scale clinical trials and an even more limited number of positive results. Future research efforts should focus on delineating the specific mechanisms of the gut microbiota and their potential applications in disease prevention and treatment, thereby providing a sound scientific basis for improving human health.
